# Javelin Head Neck 100: Should we combine immunotherapy with radiation therapy?

**DOI:** 10.18632/oncotarget.27987

**Published:** 2021-10-12

**Authors:** Yao Yu, Kaveh Zakeri, Nancy Lee

**Keywords:** immunotherapy, radiation therapy, chemotherapy, head and neck cancer, locally advanced

## INTRODUCTION

The Javelin Head Neck 100 study is the first of several randomized phase III trials to test the addition of an anti-PD1/PD-L1 antibody to chemoradiotherapy (CRT) for locally advanced head and neck squamous cell carcinomas (HNSCC) [1]. To our knowledge, it is also the first phase III trial to test immune checkpoint blockade (ICB) *concurrently* with CRT in the locally advanced setting for any disease type. In contrast to non-small cell lung cancer (NSCLC) and esophageal cancer, diseases where the addition of immune checkpoint blockade has improved outcomes in the locally advanced setting [2, 3], in Javelin Head Neck 100, avelumab failed to improve PFS and OS. This article reviews the principal results of this trial, explores comparisons to the PACIFIC and CheckMate 577 trials, and considers potential mechanistic explanations for these results. These results have implications for multiple ongoing clinical trials across many disease sites.

## JAVELIN HEAD NECK 100

In this double-blind, randomized phase III trial, patients with locoregionally advanced HNSCC were randomized to cisplatin-radiotherapy with avelumab or placebo. Avelumab or placebo was initiated one week before CRT (lead-in), then delivered concurrently with CRT, and as maintenance therapy for one year after CRT.

The trial was a superiority trial using progress-free survival (PFS) as the primary endpoint. In total, 697 patients were randomized. At the first interim analysis, the trial crossed the futility boundary and was halted. Avelumab was not associated with improvement in PFS (stratified hazard ratio 1.21, 95% CI 0.93–1.57, one-sided log-rank *p* = 0.92). Overall survival (OS) was reported in a secondary analysis and was not improved with avelumab (stratified hazard ratio 1.31, 95% CI 0.93 – 1.85, one-sided log-rank *p* = 0.94). In an exploratory subset analysis, patients with PD-L1 staining >= 25% had a trend toward improved PFS with avelumab (HR 0.59, 95% CI 0.28–1.22), and the test for interaction between treatment arm and PD-L1 staining was positive (*p* = 0.003).

Interestingly, in the patterns of failure analysis, there were more locoregional failures in the avelumab arm (37 vs. 28) but similar numbers of patients who developed distant metastatic disease (45 vs. 46). The greater number of locoregional failures in the avelumab arm, and the potential for inferior PFS and OS with avelumab, raise important questions and concerns regarding the sequencing of radiotherapy and immunotherapy.

## AVELUMAB

Avelumab is a fully-humanized anti-PD-L1 IgG approved for Merkel cell carcinoma, renal cell carcinoma, and bladder cancer. Among commonly employed anti-PD1/PD-L1 antibodies, avelumab is unique in that it has an intact Fc fragment and is capable of inducing antibody-dependent cellular cytotoxicity.

Single-agent activity with avelumab has not been reported in phase II trials in the recurrent or metastatic setting, but patients with HNSCC were included in phase I trials with avelumab [4].

## COMPARISON OF JAVELIN HEAD NECK 100 TO PACIFIC AND CHECKMATE 577: SELECTION VS. BIOLOGY

### PACIFIC and CHECKMATE 577

To date, two landmark clinical trials have integrated ICB into definitive therapy, both of which use an adjuvant immunotherapy design. In the landmark PACIFIC trial, one year of maintenance durvalumab after chemoradiotherapy improved PFS and OS for patients with locoregionally advanced NSCLC who completed definitive chemoradiotherapy [2, 5]. Post-hoc analysis of the PACIFIC trial by PD-L1 staining revealed a PFS benefit in all subgroups and an OS benefit for all subgroups except patients with tumor PD-L1 <1% [6]. In the CHECKMATE 577 study, patients treated with neoadjuvant CRT followed by esophagectomy who had residual disease on pathology were enrolled and randomized (2:1) to maintenance nivolumab vs. placebo for one year [3]. Treatment with nivolumab improved disease-free survival, thus meeting the primary endpoint of the study. The overall survival analyses have not yet been reported, pending further follow-up.

Both landmark trials incorporated ICB after completion of standard therapy, including chemoradiotherapy. Patients were required to have good performance status and laboratory values prior to enrollment. This sequential immunotherapy strategy selects patients who have good performance status and intact marrow after CRT, who are, therefore more likely to benefit from additional treatment. For example, in a retrospective study at MSKCC, up to 28% of patients who underwent chemoradiotherapy for locally advanced NSCLC were ultimately ineligible for adjuvant durvalumab [7]. Of these patients, 46% did not receive durvalumab because of persistent toxicity or concerns regarding tolerability, and 42% did not receive durvalumab due to the development of progressive metastatic disease before treatment. The design of Javelin Head Neck 100 precluded such selection. The importance of the host immune system is illustrated in a recent publication showing that the lymphocyte-to-neutrophil ratio is as predictive of response to ICB as tumor mutational burden [8].

### Impact of radiation and ICB sequencing on host biology: tumor-draining lymph nodes and depletion of host immune populations

Pre-clinical studies investigating the impact of RT and ICB sequencing on the host immune system suggest possible reasons why Javelin Head Neck 100 failed to improve outcomes, while PACIFIC and Checkmate 577 were positive.

The antitumor immune response stimulated by PD-1/PD-L1 inhibitors may be mediated by the expansion of antitumor CD8+ T cells within tumor-draining lymph nodes [9]. In definitive chemoradiotherapy, these lymph nodes are included within the elective radiation volume, where the antitumor CD8+ T cells reside. In mouse models, the inclusion of these lymph nodes leads to depletion of antitumor CD8+ T cells and abrogation of systemic antitumor immunity [9, 10]. These lymph nodes are standardly included within the radiation fields for patients with locally advanced disease.

The sequencing of ICB relative to radiotherapy may also significantly impact response to immunotherapy. Wei and colleagues investigated the impact of giving ICB before versus after a single fraction of RT (8 Gy × 1) in a mouse model. These authors demonstrated pre-radiotherapy anti-PD-1 antibody increased the radiosensitivity of CD8+ T-cells and abrogated the systemic immunity compared with mice that received ICB after radiotherapy [11]. Receipt of anti-PD-1 antibody before radiotherapy depleted the radiated and non-radiated tumors of tumor-specific CD8+ T-cells. Furthermore, anti-PD-1 given after radiotherapy resulted in better control of the radiated tumor compared with anti-PD-1 given before radiotherapy; however, combination therapy improved control over radiotherapy or anti-PD-1 therapy alone, regardless of sequence.

Because the response to radiotherapy mediated, at least in part, through the immune response [12–14], and locoregional control after radiotherapy is diminished in immunosuppressed individuals [15], selective depletion of host immune cells may be an explanation for the trends seen on the Javelin Head Neck 100 study. Supporting this hypothesis, Weiss and colleagues reported a nearly 60% risk of grade 3–4 lymphopenia in a phase II trial of pembrolizumab and concurrent radiotherapy for HNSCC, greater than would be expected with radiotherapy alone [16]. Interestingly, on flow cytometry, the authors noted a decline in CD4+ T-cells and B cells, but preserved levels of CD8+ T-cells. Tumor-intrinsic immune cells were not examined. In a phase II randomized study comparing pembrolizumab to cetuximab in combination with radiation therapy, pembrolizumab did not show improvement in locoregional control compared with cetuximab in the curative setting of locally advanced HNSCC [16, 17].

While these clinical studies are thought provoking, further analysis of biomarkers and correlatives studies from Javelin Head Neck 100 may elucidate why the study failed to improve outcomes for these patients, despite similarities between HNSCC and SCC of the lung and esophagus, diseases where ICB has established a role in definitive therapy. This work is currently ongoing and hopefully can provide further mechanistic insights.

## CURRENT ONGOING IMMUNO-ONCOLOGY TRIALS IN DEFINITIVE HNSCC

There are multiple additional phase III trials that are evaluating PD-1 or PD-L1 inhibitors in the definitive setting. (Table 1) The KEYNOTE 412 study is a phase III study, similar to Javelin Head Neck 100, that adds concurrent and maintenance pembrolizumab to definitive chemoradiotherapy for locoregionally advanced HNSCC [18]. Accrual has been completed and the study is in the follow-up phase. The REACH, NRG HN005, and NRG HN004 trials are designed with concurrent ICB and radiotherapy, with or without chemotherapy/cetuximab, followed by maintenance ICB. In contrast, IMvoke is a phase III trial of atezolizumab monotherapy after completion of definitive locoregional therapy [19], similar to the PACIFIC lung cancer trial design.

**Table 1:** Phase III trials incorporating ICB for definitive treatment of HNSCC

Phase III trials	Population	Randomization	ICB	Phase	Status	Design
Javelin HN 100 (NCT02952586)	LA HNSCC HPV−, Very High-Risk HPV+	Arm 1: Cisplatin, Radiation, Placebo Arm 2: Cisplatin, Radiation, Avelumab	Avelumab	III	Terminated, Negative	Lead-in, Concurrent, Maintenance (1 year)
KEYNOTE 412 (NCT03040999)	LA HNSCC, HPV– High-Risk HPV+	Arm 1: Cisplatin, Radiation, Placebo Arm 2: Cisplatin, Radiation, Pembrolizumab	Pembrolizumab	III	Completed accrual, outcomes not yet reported	Lead-in, Concurrent, Maintenance (14 cycles q3 weeks)
REACH (NCT02999087)	Locoregionally advanced, HPV+ or HPV− Stratified by Cisplatin eligibility	Arm 1a (Cis-eligible): Cisplatin, Radiation Arm 1b (Cis-ineligible): Cetuximab, Radiation Arm 2a (Cis-eligible): Cetuximab, Radiation, Avelumab Arm 2b (Cis-ineligible): Cetuximab, Radiation, Avelumab	Avelumab	III	Ongoing	Lead-in, Concurrent, Maintenance (1 year)
NRG HN005 (NCT03952585)	Good risk HPV+ Oropharynx	Phase II/III study. Arm 1a: Nivolumab, Radiation (reduced dose) Arm 1b: Cisplatin, Radiation (reduced dose) Arm 2: Cisplatin, Radiation (standard dose)	Nivolumab	II/III	Ongoing	Concurrent, Maintenance
NRG HN004 (NCT03258554)	Cisplatin ineligible, HPV+ and HPV−	Arm 1: Cetuximab, Radiation Arm 2: Durvalumab, Radiation	Durvalumab	II/III	Ongoing	Lead-in, Concurrent, Maintenance
Nivo PostOp (NCT03576417)	High-Risk Post-op	Post-op, ECE or SM+ Arm 1: Cisplatin, Radiation Arm 2: Cisplatin, Radiation, Nivolumab	Nivolumab	III	Ongoing	Lead-in, Concurrent, Maintenance
Atezo PostOp RTOG 1216 NCT01810913	High-Risk Post-op	Post-op, ECE or SM+ Arm 1: Cisplatin, Radiation Arm 2: Docetaxel, Cetuximab, Radiation Arm 3: Cisplatin, Atezolizumab, Radiation	Atezolizumab	II/III	Ongoing	Concurrent
IMvoke 010 (NCT03452137)	High risk of recurrence after definitive therapy (any)	Arm 1: Placebo Arm 2: Maintenance Atezolizumab	Atezolizumab	III	Ongoing	Maintenance
Pembro Neoadjuvant (NCT03765918)	High Risk Operable	Arm 1: Neoadjuvant pembrolizumab, Surgery, Post-operative Radiation +/− Cisplatin Arm 2: Surgery, Post-operative Radiation +/− Cisplatin	Pembrolizumab	III	Ongoing	Neoadjuvant, post-operative concurrent, maintenance

Abbreviations: HPV: Human Papillomavirus; LA: Locally Advanced; HNSCC: Head and neck squamous cell carcinoma; ECE: Extracapsular extension; SM+: Positive surgical margins.
